# Phenotypic and Functional Characterization of Monoclonal Antibodies with Specificity for Rhesus Macaque CD200, CD200R and Mincle

**DOI:** 10.1371/journal.pone.0140689

**Published:** 2015-10-15

**Authors:** Siddappa N. Byrareddy, Dawn Little, Ann E. Mayne, Francois Villinger, Aftab A. Ansari

**Affiliations:** 1 Department of Pathology & Laboratory Medicine, Emory University School of Medicine, Atlanta, Georgia, United States of America; 2 Department of Pharmacology and Experimental Neuroscience, University of Nebraska Medical Center, Omaha, Nebraska, United States of America; 3 Department of Microbiology & Immunology, The Yerkes National Primate Research Center, Emory University, Atlanta, Georgia, United States of America; CEA, FRANCE

## Abstract

Lectin-like molecules and their receptors are cell surface molecules that have been shown to play a role in either facilitating infection or serving as transporters of HIV/SIV in vivo. The role of these lectin-like molecules in the pathogenesis of HIV/SIV infection continues to be defined. In efforts to gain further insight on the potential role of these lectin-like molecules, our laboratory generated monoclonal antibodies (mAb) against the human analogs of rhesus macaque CD200, CD200R and Mincle, since the rhesus macaques are accepted as the most reliable animal model to study human HIV infection. The characterization of the cell lineages from the blood and various tissues of rhesus macaques that express these lectin-like molecules are described herein. Among the mononuclear cells, the cells of the myeloid lineage of rhesus macaques are the predominant cell lineages that express readily detectable levels of CD200, CD200R and Mincle that is similar to the expression of Siglec-1 and Siglec-3 reported by our laboratory earlier. Subset analysis revealed that a higher frequency of the CD14^+^/CD16^-^ subset from normal rhesus macaques express CD200, CD200R and Mincle. Differences in the frequencies and density of expression of these molecules by the gated population of CD14^+^ cells from various tissues are noted with PBMC and bone marrow expressing the highest and the mononuclear cells isolated from the colon and ileum expressing the lowest levels. While a significant frequency of pDCs and mDCs express Siglec-1/Siglec-3, a much lower frequency expresses CD200, CD200R and Mincle in PBMCs from rhesus macaques. The mAb against CD200 and CD200R but not Mincle appear to inhibit the infection of macrophage tropic SIV/SHIV in vitro. We conclude that these mAbs may have potential to be used as adjunctive therapeutic agents to control/inhibit SIV/HIV infection.

## Introduction

While the CD4 molecule in association with CCR5 and CXCR4 the 2 major co-receptors are known to play critical roles in the entry of HIV and SIV into CD4^+^ T cells, it is gradually being recognized that a variety of additional molecules that include the lectin-like receptors (LLRs), integrins such as α4β7 and receptors for lipid associated proteins may also play an important role either in the transport of the virions or facilitating their entry into the cells [1]. Both the HIV and SIV *env* are heavily glycosylated comprising more than 20% of the constituents of the virus and thus reasoned to utilize such glycosylated residues to bind to cells that express receptors against such molecules [2]. The glycans that decorate the envelopes of the viruses are synthesized in the endoplasmic reticulum (ER) and Golgi complex involving enzymes of the host cell glycosylation pathways [3, 4]. These views have led to the study of glycosylation deficient recombinant SIVmac239 as tools to define the role of *env* glycosylation in virus-host interactions [5]. The fact that such glycans can a) serve either as a shield to prevent recognition of critical immunogenic sequences of the virions by the host immune system [6, 7] or prevent the induction of protective immune responses against the protein backbone of the virus; b) influence the selective transmission of “founder” viruses in HIV-1 infection [8–11]; and c) contribute to the relative pathogenicity of the virus as shown by the study of recombinant SIVmac239 that lack critical glycosylated *env* residues [12–14], has heightened interest in the characterization of such glycans with obvious implications for advances in vaccine design and formulations. Thus, not only do the HIV/SIV *env* become glycosylated within the lumen of the ER and golgi resulting in greater that 25 residues that become glycosylated but, in addition, during the budding process from the membrane of the host cells the virions acquire host cell glycoproteins which together serve to shield the virions from host cell immune responses [15, 16]. There are basically two forms of N-glycans that have so far been characterized as part of the glycans that decorate the HIV/SIV virions. These include the oligo-mannose type glycans that comprise approximately 70% of the glycans and the complex type glycans that constitute the remaining 30% of the glycans [6]. These glycans occur in homogeneous patches with the complex glycans occurring proximal to the CD4 binding site and the oligo-mannose expressed distal to the CD4 binding site of the viral envelope [17]. There is also a high degree of glycan-glycan interactions that result in tight clusters that are thought to contribute to facilitate interaction with the corresponding cognate lectin-like receptors on host cells. Details of the studies of the structure, function and biology of C-type lectin receptors have been summarized elsewhere [18]. In addition, how such glycan-lectin interactions influence the progress of HIV-1 infection [19] and viral infections in general that can either benefit the host or the virus have been a subject of a previous review [20].

The importance of HIV/SIV glycans and the lectin-like host cell receptors outlined above, prompted us to initiate studies aimed at defining the role of such lectin-like receptors in the non-human primate (NHP) model of HIV-1 infection. Towards this end, our laboratory was the first to characterize the differences in the expression of a series of Siglec molecules by cells from disease resistant NHP natural hosts of SIV and their disease susceptible non-natural NHP hosts [21]. We have now extended these studies and herein include the characterization of the expression and potential function of select additional lectin-like receptors that have been shown to play an important role in regulating inflammation as part of both the innate and adaptive immune responses [22] and those involved in distinguishing self from non-self and have the capacity to bind a wide range of carbohydrates. These molecules include the rhesus macaque analogs of human CD200, CD200R and Mincle. Their expression and initial functional characterization by cells from non-human primates (NHP) that serve as models of human HIV-1 infection is the subject of this communication. It is important to note that CD200 has recently been identified as a marker uniquely expressed by T follicular helper (Tfh) cells [23]. In addition, it was our objective to determine whether such receptors are differentially expressed by cells from sooty mangabeys, the disease resistant natural NHP hosts of SIV (as we have previously described (21), as compared with cells from rhesus macaques that are susceptible to SIV infection and disease [24]. Results of such studies are the basis of this report.

## Results

### Identification of the hematopoietic cell lineage(s) that express CD200, CD200R and Mincle

Our interest in characterizing the role of lectin-like receptors in the pathogenesis of HIV/SIV infection prompted us to prepare a series of monoclonal hybridoma cell lines that synthesized antibodies against rhesus macaque analogs of human CD200, CD200R and Mincle. We initially screened the reactivity of the monoclonal antibodies (mAbs) using the 2 human monocytic cell lines (U937 and THP-1) and subsequently freshly isolated PBMC samples from normal healthy rhesus macaques and humans. We also utilized the Siglec-1 and Siglec-3 specific monoclonal antibodies that we had previously characterized [21] for purposes of positive control and to serve as a standard.

**Fig 1:**
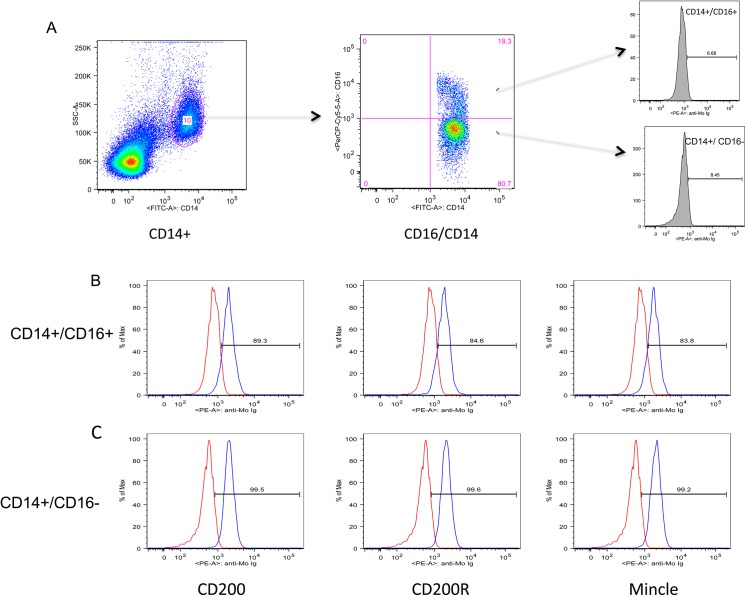
The gating strategy utilized for the analysis of CD14^+^ cells and the 2 subsets that showed significant profiles that include the CD14^+^/CD16^+^ cells and the CD14^+^/CD16^-^ population in PBMCs of healthy rhesus macaques is displayed (panel A). These 2 subsets were then analyzed for the frequency and mean fluorescent intensity (MFI) following staining with monoclonal antibodies with specificity for rhesus macaque CD200, CD200R and Mincle (panel B & C). The red profile reflects the anti-mouse Ig control and the blue profile reflects the expression profile of CD200, CD200R and Mincle, respectively.

Studies were first performed to identify the hematopoietic mononuclear cell lineage(s) in the blood from rhesus macaques that express CD200, CD200R and Mincle and for purposes of reference other lectin-like molecules such as Siglec-1 and Siglec-3 previously characterized by our lab [21]. The predominant mononuclear cell population that expressed these lectin-like molecules was identified as cells of myeloid origin with the CD14^+^ cells exhibiting the most distinct profile (Fig 1A). Two of the subsets of CD14^+^ cells expressed readily detectable levels of these molecules. These included the CD14^+^/CD16^-^ cell lineage defined as classical monocytes (comprising > 70% of the total monocyte population) and the CD14+/CD16+ intermediate population (comprising 14–15% of the total population of monocytes) [25]. In general, the CD14+/CD16- subset expressed higher mean densities of CD200, CD200R and Mincle (Fig 1B & 1C). The analysis of data obtained on the expression of CD200, CD200R and Mincle by the CD14dim/CD16+ subset was highly variable results for reasons unclear and were therefore not studied further. In addition, due to their myeloid origin, the plasmacytoid and the myeloid dendritic cells (pDCs and mDCs) were also examined and the results reported below. The other hematopoietic cell lineages (such as T cells, B cells and NK cells) showed either low densities of expression or too low frequencies (0.5–1% of each lineage) that constitutively express these lectin-like molecules and their study was therefore not pursued further. Thus, in all the studies described below, our analysis of CD200, CD200R and Mincle expressing cells was focused on the gated population of CD14 expressing cells in PBMC samples (S1 Fig) and on CD14 expressing cells isolated from a variety of other tissues.

**Fig 2:**
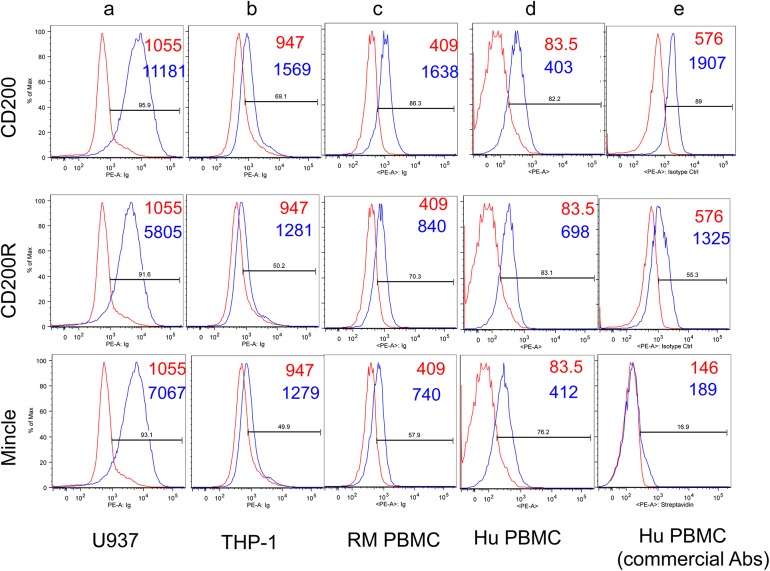
Representative flow cytometric profiles of CD200, CD200R, Mincle, Siglec-1 and Siglec-3 expression by the U937 (panel a) and THP-1 (panel b) cell lines and by the gated population of CD14+ cells from PBMCs from a representative normal rhesus macaque (panel c) and for comparison the profile of the gated population of CD14+ cells from the human PBMCs stained with our panel of anti-CD200, CD200R and Mincle antibodies (panel d) and the commercially obtained monoclonal antibodies with specificity for human CD200, CD200R and Mincle (panel e). The profile in red reflects the use of anti-mouse Ig (control) and the one in blue reflects the profile noted with the appropriate anti-lectin monoclonal antibody (mAb). The numbers in red and blue are the MFI values noted for the anti-mouse Ig control and the anti-lectin mAb, respectively.

As seen in Fig 2 (panels a and b), each of the monoclonal antibodies against CD200, CD200R, and Mincle showed a higher mean fluorescent intensity (MFI) on the U937 cell line as compared with the THP-1 cell line (please note that the red lines and numbers represent the profile and MFI of the isotype control and the blue line and numbers the reactivity and MFI of the appropriate monoclonal antibody being evaluated in each of the figures). The reactivity of the same monoclonal antibodies on the gated population of CD14+ cells from the PBMCs of a representative normal healthy rhesus macaques (n = 16) and humans (n = 3) showed readily detectable levels of CD200, CD200R and Mincle on resting PBMCs (Fig 2, panels c and d). Use of commercially available monoclonal antibodies against CD200, CD200R and Mincle was also analyzed for purposes of comparison and the representative profiles are shown in Fig 2, panel e. The commercially available anti-Mincle monoclonal antibody stains poorly on resting human PBMCs but reacts reasonably well using in vitro activated monocytes (data not shown). Since Siglec-1 and 3 are expressed by the monocytic cells, we also analyzed their expression on rhesus PBMC’s and the U937 and THP-1 cell line for purposes of comparison (S2 Fig). Of interest was the finding that while the THP-1 cell line expressed significantly lower levels of CD200, CD200R and Mincle this cell line expressed readily detectable levels of both Siglec-1 and Siglec-3. It is important to note that human monocytes do not express detectable levels of Siglec-1 [21]. The reason for the high levels of Siglec-1 expression by the U937 cell line remains unclear although this cell line is considered less differentiated than the THP-1 cell line.

**Fig 3:**
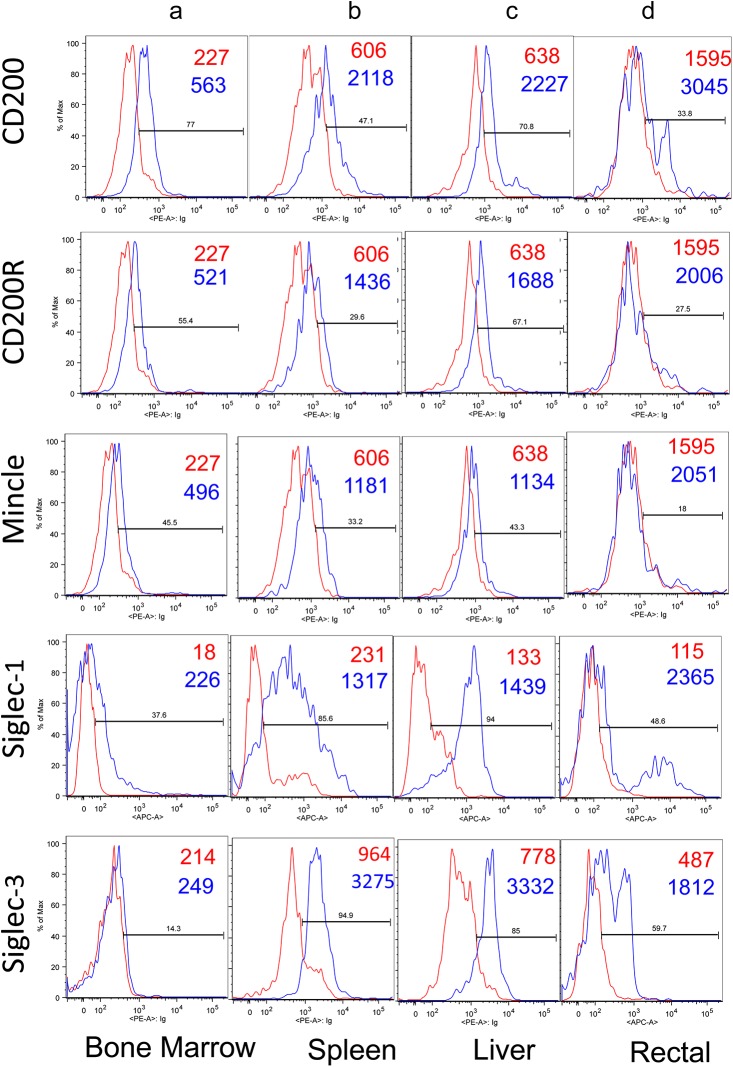
Representative flow cytometric profile of CD200, CD200R, Mincle, Siglec-1 and Siglec-3 expression on the gated population of CD14^+^ cells isolated from the bone marrow (panel a), spleen (panel b), liver (panel c) and mononuclear cells isolated from rectal biopsy samples (panel d) from a representative healthy normal rhesus macaque. The profile in red reflects the use of anti-mouse Ig (control) and the one in blue reflects the profile noted with the appropriate anti-lectin monoclonal antibody (mAb). The numbers in red and blue are the MFI values noted for the anti-mouse Ig control and the anti-lectin mAb, respectively.

We next examined the relative density profiles of these same lectin-like molecules on the gated population of CD14^+^ cells from various tissues of rhesus macaques. Thus, as seen in Fig 3 (panel a) while the gated population of CD14^+^ bone marrow cells clearly expressed CD200, CD200R and to a lower extent Mincle, they appear to express low levels of Siglec-1 but no detectable level of Siglec-3 expression. The CD14^+^ cells from the spleen and liver also showed readily detectable levels of CD200, CD200R, Siglec-1 and Siglec-3 but comparatively lower levels of Mincle (Fig 3, panels b and c). The CD14^+^ cells isolated from rectal biopsy samples showed relatively low-density profiles for CD200 and CD200R (Fig 3, panels d) but reasonable levels of expression of Siglec-1 and Siglec-3 but barely detectable levels of Mincle.

When the frequencies of CD14^+^ cells from various tissues were analyzed (please do note that differences do exist in the MFI noted for each of these molecules which may play a role in the function of these molecules by each cell lineage), there appeared to be in general, a hierarchy, in the frequencies of CD14^+^ cells that express CD200, CD200R and Mincle with the highest levels for CD200 followed by CD200R and Mincle (Table 1). Similarly, there was a hierarchy in the frequencies in the CD14^+^ gated population of cells from the various tissues that expressed CD200, CD200R and Mincle with PBMC’s showing the highest value followed by bone marrow, liver, spleen, lymph node cells and among the CD45^+^ cells isolated from rectal biopsy tissues. The lowest frequencies were noted in cells isolated from the colon and ileum tissues (Table 1). With regards to Siglec expression, it was interesting to note that while there was a low frequency of Siglec-3 expressing CD14^+^ cells obtained from rectal biopsy specimens, the gated population of CD14^+^ cells from the liver had very high levels of Siglec-3 expressing cells.

**Table 1:** Comparative analysis of the frequencies of CD14+/CD16- cells expressing CD200, CD200R, Mincle, Siglec-1 and Siglec-3 in different tissues from rhesus macaques and sooty mangabeys.

Species of Monkey/tissues	Numbers (n)	CD200	CD200R	Mincle	Siglec-1	Siglec-3
				
Rhesus Macaques						
PBMC-adult	16	83.5 ± 7.0	66.7 ± 18.0	52.5 ± 20.8	52.0 ± 24.5	79.7 ± 14.0
Rectal cells	5	29.6 ± 19.5	18.5 ± 14.0	18.0 ± 18.0	36.5 ± 7.0*	11.0 ± 3.8*
Spleen	6	56.3 ± 5.0	36.1 ± 6.0	28.0 ± 2.7	62 ± 9.4*	24.7 ± 46.0*
Bone marrow	8	58.2 ± 16.4	50.2 ± 11.9	46.5 ± 12.1	38.7 ± 9.9*	45.1 ± 37.7*
Liver	7	51.5 ± 2.4	38. 6 ± 1.0	33.6 ± 5.5	54.9 ± 4.7*	74.1 ± 1.0*
Colon	5	7.0 ± 5.0	3.0 ± 5.0	5.5± 6.7	ND	ND
Ileum	3	6.8 ± 4.0	6.8 ± 4.7	7.1 ± 4.7	ND	ND
Lymph node cells	5	26.6 ± 17.7	19.5 ± 13.6	19.3 ± 14.8	ND	ND
Sooty Mangabeys						
PBMC-adult	26	22.0 ± 12.4	9.3 ± 6.6	7.8 ± 4.8	42.4 ± 16.0	98.5 ± 3.2
Rectal cells	10	ND	ND	ND	30.0 ± 14.0	7.5 ± 11.5

* Represents n = 2 samples were analyzed for Siglec-1 and Siglec-3; ND, not determined.

**Fig 4:**
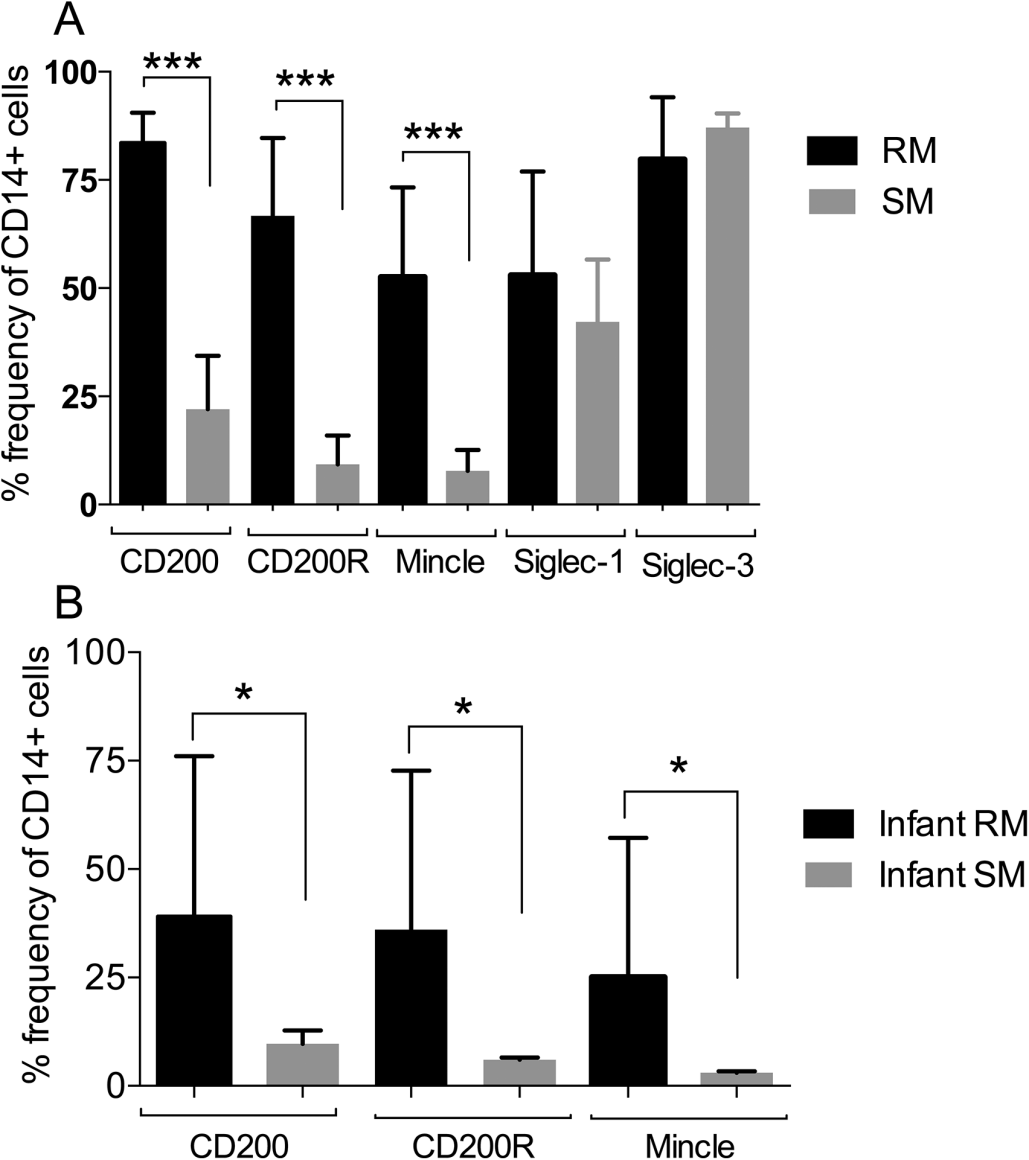
Comparative analysis of the frequencies of CD200, CD200R, Mincle, Siglec-1 and Siglec-3 expressing cells by the gated population of CD14^+^ cells from the PBMC samples from normal adult rhesus macaques (n = 19, black bars) and sooty mangabeys (n = 18, grey bars) (A) and similar analysis on infant (< 3 months) rhesus macaques (n = 10) and sooty mangabeys (n = 4) (B). The notations * represents p < 0.05 and *** represents p < 0.001.

We also compared the relative expression of the lectin like molecules on the gated population of CD14^+^ cells in PBMC samples from the natural hosts of SIV, the disease resistant sooty mangabeys as compared with the disease susceptible rhesus macaques. The sooty mangabeys share 96.5 (CD200), 97.8 (CD200R) and 92.3% (Mincle) homology at the nucleotide level with rhesus macaques. As seen in Fig 4A, the gated population of CD14^+^ cells from sooty mangabeys showed markedly lower levels of CD200, CD200R and Mincle expression as compared with the same population from RM (we recognize that these differences could reflect differences in the binding efficiencies of these molecules from the 2 species). On the other hand, the CD14^+^ cells from sooty mangabeys expressed essentially similar frequencies of Siglec-1 and Siglec-3 as compared with RM. In efforts to determine whether the differences in CD200, CD200R and Mincle expressing CD14^+^ cells from the 2 species were age related, we examined PBMC’s from < 3-month old infants from these 2 species. Basically, while the CD14^+^ cells from infant RM showed a much lower frequency of CD200, CD200R and Mincle expressing cells as compared to adult RM, the CD14^+^ cells from the PBMCs of SM continued to show significantly lower frequencies than infant RM (Fig 4B). Of interest, there was no detectable difference in the frequencies of CD14^+^ cells from infant RM and SM that expressed Siglec-1 and Siglec-3 as compared with CD14^+^ cells from adult animals of the same species (data not shown).

**Fig 5:**
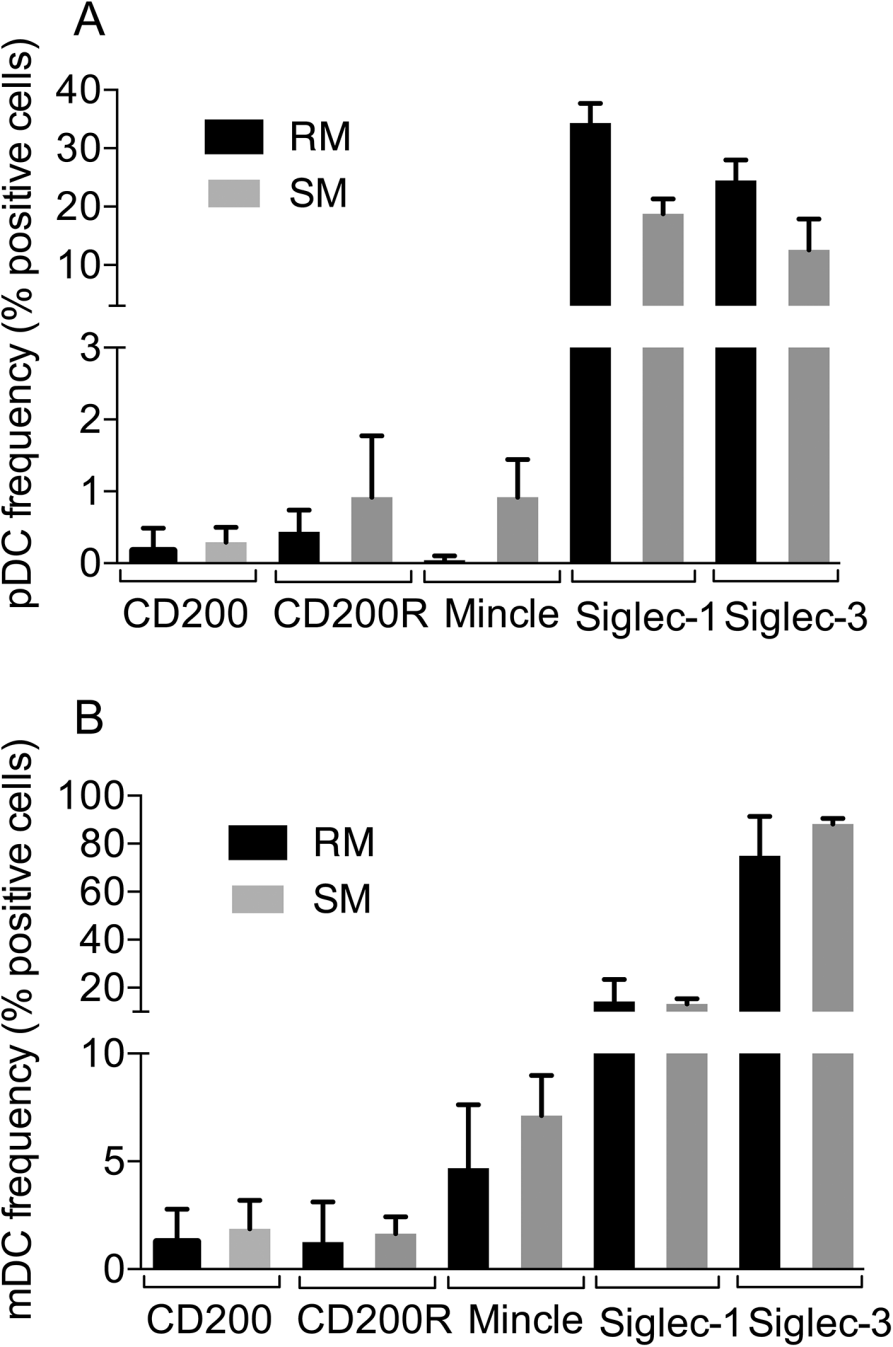
The net frequencies (background using isotype control Ig deducted) of CD200, CD200R, Mincle, Siglec-1 and Siglec-3 expressing Lin^-^, HLA-DR^+^, CD123^+^ plasmacytoid dendritic cells (pDCs, panel A) and Lin^-^, HLA-DR^+^, CD11c^+^ myeloid dendritic cells (mDCs, panel B) in the peripheral blood samples from 3 normal rhesus macaques and 3 normal sooty mangabeys are displayed. The absolute numbers of mDCs noted in the blood of rhesus macaques were 355 +/- 42 and pDCs were 48 +/- 17. The data reflect the Mean +/- SD values and * reflects p< 0.05, ** reflects p< 0.01.

We next examined the frequencies of mDCs and pDCs in blood samples from these 2 species for the expression of CD200, CD200R and Mincle. The gating strategy for identifying the frequencies of CD200, CD200R and Mincle expressing mDCs and pDCs is shown in S3 Fig As seen in Fig 5A & 5B, while a very low frequency of pDCs and mDCs expressed CD200 and CD200R, there appeared to be a slightly higher frequency of mDCs that expressed Mincle than the pDCs although the differences were not statistically significant. Of interest, both the pDcs and mDCs expressed significantly higher levels of Siglec-1 and Siglec-3.

### Effects of anti-CD200, CD200R and Mincle on SHIV/SIV infection in vitro

**Fig 6:**
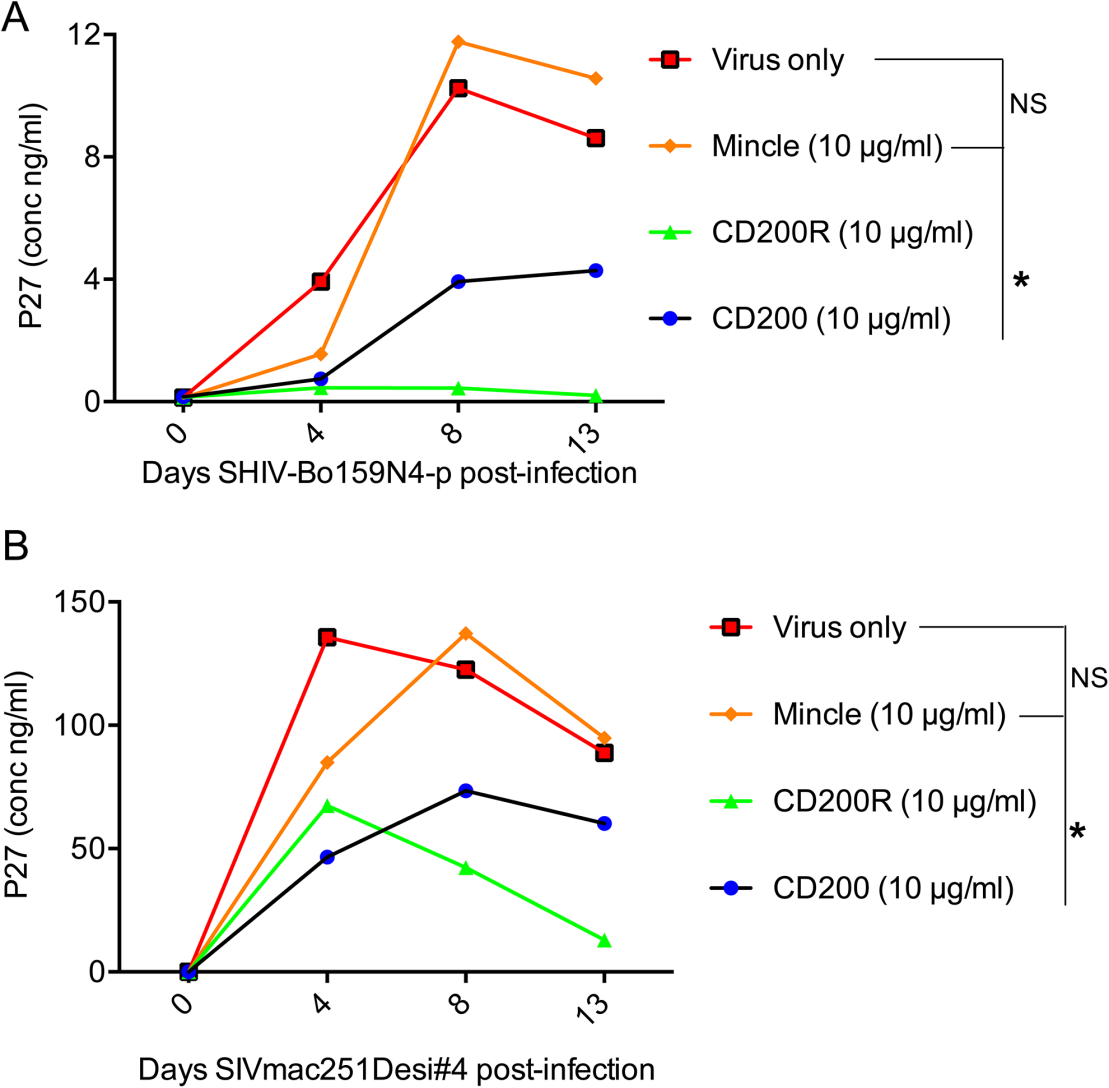
Effect of anti-CD200/CD200R/Mincle monoclonal antibodies on the in vitro replication of macrophage tropic SHIV. Monocytes were isolated using a procedure outlined in the methods section. A 48 well plate was seeded with 10^4^ monocytes/well and cultured for 7 days and aliquots infected with either SHIV-Bo159N4 (A) or SIVmac251Desi#4 in the presence of normal mouse Ig (10 μg/ml) or 10 μg/ml of the monoclonal antibodies against either CD200, CD200R, or Mincle. Culture supernatants were collected at various time points as shown and the p27 levels quantitated using a commercial ELISA kit. NS = non-significant and the notation * reflects a ‘p’ value of <0.05 noted for data obtained with the addition of mAb against CD200 or CD200R.

We next examined the potential of the mAbs against these lectin-like molecules expressed by CD14^+^ cells to influence SIV infection and replication in vitro using primary cultures of monocyte derived macrophages from RM as described in the methods section. We utilized a stock of the highly macrophage tropic SHIV-Bo159N4-p [26] and desiv147#4 [27] viruses prepared in our lab for these studies. We incubated aliquots of the target cells with various concentrations (1, 5 and 10 μg/ml) of the anti-CD200, CD200R, Mincle and for purposes of control normal mouse IgG for 2 hrs and subsequently infected the cultures with 10^5^ TCID_50_ of each of the 2 viruses. As shown in Fig 6A & 6B, at 10 μg/ml both anti-CD200 and CD200R significantly (p<0.05) inhibited virus replication as compared to cultures incubated with 10 μg/ml of control IgG and virus only. The mAb against CD200R was more effective showing >70% inhibition as compared to virus only treated cells. However, the anti-Mincle mAb at the concentration utilized failed to show any detectable effect. The inhibitory effects of the mAbs with specificity for CD200 and CD200R was specific since aliquots of the same mAbs (10 μg/ml) when pre-incubated with their corresponding immunodominant target peptide (5 μg/ml) prior to assay for virus inhibition as described in the methods section (but not irrelevant peptide also at 5 μg/ml) failed to show inhibition of virus replication (data not shown). Collectively these data indicate that the CD200/CD200R lectin-like molecules must play a role in the susceptibility of cells to infection against at least the viral isolates utilized. Further studies are in progress with the objective to define the mechanisms including the stage of virus infection by which these antibodies influence virus infection in vitro.

## Discussion

It is clear from the results of the studies reported herein that the predominant cell lineage that express CD200, CD200R and Mincle is the monocyte/macrophage cell lineage. These data are consistent with a number of previous studies that have shown that lectin-like molecules and their cognate receptors are to a large extent expressed by cell lineages that are part of the innate immune system. These include the well studied Ly49 receptors [28] the dectin-1/dectin-2 family that includes Mincle [18, 29] ficolins [30] and the variety of pattern recognition receptors [31]. The fact that such lectin-like molecules are also expressed on NK cells that primarily control cellular activation by recognition of peptide bearing MHC class I and related molecules suggests that their function on NK cells is likely to be relatively restricted as compared with the macrophage and dendritic cells that have a much more diverse set of function and ligands [32]. The fact that considerable heterogeneity of the monocytes/macrophage lineages exists within the blood prompted us to determine whether the expression of CD200, CD200R and Mincle showed differences in the frequencies and/or density of expression by subsets of monocytes/macrophages. Fig 1A, 1B and 1C shows the profiles seen for the CD14^+^/CD16^-^ and the CD14^+^/CD16^+^ subset of cells that constitutively express these lectin-like molecules. Of interest, the CD14dim/CD16^++^ subset did not appear to show consistent display of the CD200, CD200R and Mincle molecules for reasons unclear at present. Attempts to determine if in vitro activation of the monocytes with appropriate activating agents (LPS, TNF-alpha, IL-6, IFN-gamma) may induce increased levels of expression of these molecules failed to show any significant differences (data not shown) suggesting that their expression is not linked to the pathways associated with cell activation. Similarly, preliminary studies utilizing blood samples from SIV infected rhesus macaques during chronic infection failed to show any detectable difference in the frequencies and densities of CD200, CD200R and Mincle on the CD14^+^ gated population of cells (data not shown). This failure to induce increased expression of CD200, CD200R and Mincle is in contrast to the effects of activation agents and SIV infection on the expression of Siglec-1 and Siglec-3, where significant up-regulation has been previously documented [33]. These findings suggest that the signaling pathways that regulate the expression of CD200, CD200R, and Mincle are distinct from those that regulate the expression of Siglec’s.

We also examined the frequencies and density of expression of CD200, CD200R and Mincle by the gated population of CD14^+^ cells from a variety of tissues from rhesus macaques since heterogeneity of this cell lineage has been shown to be dictated by the tissue micro-environment in which this lineage resides [34]. While some differences were noted, there did not appear to be any major difference in the level (MFI) of expression of CD200, CD200R and Mincle on at least the CD14^+^ cell lineage in these various tissues (Fig 3). In contrast, there did appear to be significant differences in the density of Siglec-1 and Siglec-3 expressed by cells from these various tissues. When the frequencies of CD14^+^ cells that express these molecules were examined, however, there appeared to be some distinct differences (Table 1). Thus, the CD14^+^ cells from the PBMCs, spleen and liver appear to have the highest frequencies of CD200, CD200R and Mincle expressing cells followed by mononuclear cells isolated from rectal biopsy tissues and lymph node cells. The lowest frequency of CD200, CD200R and Mincle expressing CD14^+^ cells were noted in the cells from the colon and ileum (Table 1). Further studies utilizing other markers that distinguish the monocyte/macrophage subsets [35, 36] are needed to examine this issue in more detail. This is in fact true for tissue derived macrophages that are now known not to be all derived from hematopoietic precursors and that they are not all terminally differentiated [37]. Interests in the heterogeneity of macrophage sub-types has been generated by studies of atherosclerosis that have documented the existence of the Th1-polarized pro-inflammatory classical M1 macrophages and the Th2-polarized anti-inflammatory M2 macrophages that contribute to tissue remodeling with further sub-types identified as M2a, b, c and d [38] A lack in the availability of rhesus macaque reactive monoclonal antibody reagents for other cell surface markers and cell lineages and a difficulty in obtaining fresh tissue specimens (since the macrophage lineages do not all survive cryopreservation) specimens from internal organs of monkeys are some of the limitations to address this issue.

The other cell lineages that were noted to express readily detectable levels of CD200, CD200R and Mincle are the dendritic cell lineages that are also heterogeneous [39] in nature and the data need to be interpreted with this heterogeneity in mind. The results of our studies, although preliminary, showed that in fact a very low frequency of the gated population of pDCs and mDCs constitutively expressed CD200 and CD200R (Fig 5A & 5B). There did appear to a slightly higher frequency of mDCs than pDCs that expressed Mincle but the significance of this difference is not clear at present. The pDCs and mDCs are relatively resistant to HIV and SIV infection in vitro due to the presence of significantly high levels of the restriction factor SAMHD1 [40]. While the DCs are resistant to infection, HIV-1 and presumably SIV infection does induce dysfunction of the DCs [41] by mechanisms that have yet to be completely defined. We submit that these lectin-like receptors must not play a major role in such dysfunction due to the low frequency of this cell lineage that expresses these molecules. The data presented herein we submit, however, provides a foundation for future studies that involve the function of these lectin-like molecules by these cell lineages in specific disease conditions. It is also important to note that the sooty mangabeys that are the natural hosts of SIV and are disease resistant appear to show markedly lower levels of these lectin-like molecules expressed by pDCs and mDCs (Fig 5A and 5B). However, it is quite possible that the reagents we utilized may not be optimal for the detection of the sooty mangabey analogs of the rhesus macaque CD200, CD200R and Mincle.

Since the data reported herein involved studies of the CD200, CD200R and Mincle molecules, it is reasoned that a brief discussion of the role of these molecules is deemed appropriate. The pair of CD200 and CD200R molecules is gaining increasing attention lately due to the finding that the interaction between these two molecules induces a suppressive and a negative regulatory effect in both peripheral and mucosal immune responses much like the ligation of PD-1 with its cognate receptor PD-L1 specially against viral infections [42]. As a matter of fact, blocking of this negative regulatory function is currently being exploited for the therapy of patients with AML [43] and has been shown to be involved in various models of transplant allograft survival [44]. In lieu of this finding, it is tempting to speculate that such blocking function should be considered as a therapeutic tool to enhance SIV and in the future HIV specific immune responses.

The macrophage C-type lectin (MCL) and macrophage inducible C-type lectin (Mincle also termed Clec4e or Clecsf9) have been shown to be a part of the varied numbers of pattern recognition receptors that have the capacity to recognize damaged autologous cells and pathogen-associated molecular patterns (PAMPs). The carbohydrate recognition domain (CRD) of Mincle has been shown to mediate the recognition of PAMPs [45]. The ability to recognize damaged cells has been thought to be part of the ability of immune cells to distinguish self from non-self and thus a role in tolerance [46]. The role of Mincle and MCL in bacterial and fungal infections has been well documented include studies of structure-activity relationships with ligands that include the trehalose dimycolate and other mycobacterial glycolipids. These interactions have been shown to influence the development of Th1/Th17 type of immune responses and thus considered important for the formulation of vaccine strategies [47]. The frequencies and densities of Mincle expressed by the CD14 gated population of cells was clearly much less than the CD200 and CD200R molecules and once again, no significant differences were noted when the same population was examined from chronically SIV infected rhesus macaques.

One of the most interesting findings from the studies reported herein concern the ability of the monoclonal anti-CD200 and CD200R to inhibit SHIV infection in vitro using monocyte derived macrophages (Fig 6). One of the issues with regards to using such monocyte derived macrophages is whether such in vitro derived macrophages following derivation still retain their expression of CD200, CD200R and Mincle. We did examine the expression of CD200, CD200R and Mincle on in vitro matured monocyte derived macrophages. The data showed that while there was a decrease in the MFI for each of these molecules (S4 Fig) there was still significant levels of at least CD200 and CD200R expressed. The fact that that the addition of mAb’s against these molecules showed biological activity suggests that even at decreased levels of expression, the levels are sufficient to affect biological function. It is also clear that the failure of the anti-Mincle mAb to show biological effect could be due to the diminution in the level of Mincle expressed by the in vitro matured macrophages. The findings of the anti-viral effect are in some regard counter intuitive since the ligation of CD200 with its natural ligand CD200R is thought to induce negative regulatory signals and create an intra-cellular immunosuppressive environment that would promote viral replication. The finding that some viruses including the herpes viruses including the rhesus macaque rhadinovirus [48] encode functional orthologs of CD200 and use the CD200/CD200R pathway to establish chronic infections [42] is a good example of the above view. It is possible that the physiological interaction between CD200 and CD200R involves molecular motifs that are distinct from those recognized by the monoclonal antibodies utilized in the present studies. The monoclonal antibodies may augment the negative signaling pathway leading to an environment that is not favorable for lentiviral replication. Clearly, further studies are required to define the mechanisms by which the mAbs against CD200 and CD200R mediate their inhibitory effect of SIV/SHIV replication in vitro and such studies are in progress.

## Materials and Methods

### Ethics Statement

The animals included Indian origin rhesus macaques and African origin sooty mangabeys that were bred in the nonhuman primate breeding facility of Emory University, Atlanta, Georgia, USA. The animals from which blood samples and tissues were obtained utilized in the present study were born and maintained at the Yerkes National Primate Research Center (YNPRC) of Emory University, Atlanta, Georgia, USA. Their maintenance was performed in accordance with the rules and regulations of the Committee on the Care and Use of Laboratory Animal Resources of the U.S. Public Health Service/National Institutes of Health, as well as according to the recommendations outlined in the Weatherall report on “The Use of Non-human Primates in Research” (http://www.acmedsci.ac.uk/images/project/nhpdownl.pf). The animals were fed a monkey diet (Purina, Gray Summit, MO) supplemented daily with fresh fruit or vegetables and water ad libitum. Additional social enrichment including the delivery of appropriate safe rubber toys, access to perches, varied treats such as peanuts and cereals that were provided and overseen by the Yerkes enrichment staff and animal health was monitored daily and recorded by the animal care staff and veterinary personnel, available 24/7. Monkeys were caged in socially compatible same sex pairs in a temperature controlled (maintained at 72F) and 12-hour light/dark cycle light controlled indoor facility to facilitate social enhancement and well being. Monkeys were closely monitored and observed for development of distress or disease twice daily. Blood was collected under ketamine or Telazol anesthesia from the femoral vein. Animals showing signs of sustained weight loss, disease or distress were subject to clinical diagnosis based on symptoms and then provided either standard dietary supplementation, analgesics and/or chemotherapy as directed by the veterinary staff. Monkeys with sustained weight loss whose symptoms could not be alleviated using standard dietary supplementation, analgesics and/or chemotherapy were humanely euthanized using an overdose of barbiturates according to the guidelines of the American Veterinary Medical Association. None of the monkeys that served as donors of blood or tissue samples were sacrificed for the purpose of this study. Some of the tissues utilized were obtained at autopsy from animals that were sacrificed during the course of other studies when they reached the end points (described above) of the respective study by procedures outlined above. The studies reported herein were performed under IACUC protocol #2001725 "Gut Homing Cells in SIV Infection" which was reviewed and approved by the Emory University IACUC. It has been assigned the IACUC protocol number "YER-2001725-042715GA". The YNPRC has been fully accredited by the Association for Assessment and Accreditation of Laboratory Animal Care International since 1985. In addition, all experiments were reviewed and approved by the Emory biosafety review Committee prior to initiation of the studies.

### Source of blood samples and tissues

We obtained heparinized blood samples and a variety of tissues from rhesus macaques (Macaca mulatta), and sooty mangabeys (Cercocebus atys) housed at the Yerkes National Primate Research Center (YNPRC) of Emory University. The nonhuman primates belonged to cohorts of animals being studied by us on various protocols (IACUC protocol #2001725 “Gut homing cells in SIV infection),” or as specimen requests from the YNPRC. The housing, care, diet, and maintenance conformed with the guidelines of the Committee on the Care and Use of Laboratory Animals of the Institute of Laboratory Animal Resources, National Research Council, and the Department of Health and Human Services guidelines titled Guide for the Care and Use of Laboratory Animals. The Emory Institutional Animal Care and Use Committee approved the entire study including the collection of blood and rectal biopsies prior to the initiation of these studies. The YNPRC has been fully accredited by the Association for Assessment and Accreditation of Laboratory Animal Care International since 1985. All the animals were negative for SIV, simian T cell lymphotropic virus, and simian retrovirus.

### Isolation of cells from blood and tissues

Mononuclear cells were isolated from rhesus macaque and human PBMC and colorectal biopsy specimens from rhesus macaques as described previously [49]. In brief, heparinized blood samples were obtained and mononuclear cells were isolated using ficoll-hypaque gradient centrifugation. A series of 12–16 pinch colorectal biopsies/sample from each of the species of monkeys were subjected to a series of purification steps and finally isolated by Percol gradient and isolated using standard techniques as described previously [49]. The yields and viability of the pooled cells extracted from the biopsy tissues varied considerably; the viability ranged from 62 to 84% as determined by trypan blue dye exclusion method. In addition, specimens of bone marrow, spleen and liver were obtained at autopsy from rhesus macaques as part of a number of other protocols being carried out by our laboratory. The tissues were minced and a single cell suspension from these tissues placed on a Ficoll-hypaque gradient and following centrifugation at 450 X g, the cells at the interface collected, washed and re-suspended in RPMI media supplemented with 1% antibiotics, 2 mM L-glutamine and 10% heat inactivated fetal calf serum (complete RPMI media). Since the predominant expression of these lectin-like molecules were found to be cells of the monocyte lineage, we utilized the U937 and THP-1 cell lines as potential references for standardization of the data obtained on primary cells from the two NHP species.

### Antibodies

The studies conducted utilized both a variety of nonhuman primate reactive commercially available monoclonal antibodies and a series of monoclonal antibodies prepared by our laboratory. The commercially available monoclonal antibodies included PerCP-Cy5.5-CD16, Cat# 338426 (Clone 3G8; BD); FITC-CD14, Cat# 557153, (Clone M5E2; BD); Goat Anti-Mouse Ig, Human ads-PE, Cat# 1010–09, (Isotype IgG, Southern Biotech); APC-CD169 (Siglec-1), Cat# 17-1699-42, (Clone 7–239; eBioscience); PE-anti-h-Siglec-3, Cat# FAB1137P, (Clone 6C5/2: R&D); Mouse PE-IgG1 Isotype Control, Cat# IC002P, (Clone 11711, R&D); FITC-CD3, Cat# 556611, (Clone SP34, BD); FITC-CD8, Cat# 347313 (Clone SK1; BD); FITC-CD11b, Cat# 1M0530U, (Clone Bear 1; Beckman Coulter); FITC-CD20, Cat# 556632, (Clone 2H7; BD); APC-CD11c, Cat# 340544, (Clone S-HCL-3; BD); PerCP-Cy5.5-HLA-DR, Cat # 552764 (Clone G46-6: BD); PerCP-Cy5.5-CD123, Cat# 55871, (Clone 7G3: BD); PE-Texas Red-HLADR, Cat# MHLDR17, (Clone Tu36: Life Technologies); PE-anti-human CD200 (clone OX-104, Biolegend), PE-anti-human CD200R (clone OX-108, Biolegend), and PE-anti-human Mincle (clone 15H5, Biolegend). These fluorochrome-conjugated antibodies were utilized at a pre-determined optimum concentration as recommended by the manufacturer. Polychromatic flow cytometry was performed on stained cell samples using a variety of combination of these antibodies that identified monocytes and plasmacytoid/myeloid dendritic cells.

In addition, our lab has generated a series of murine monoclonal antibodies against rhesus analogs of human CD200, CD200R and Mincle (prepared by Abmart, Shanghai, China on contract) and selected those with the highest affinity and specificity for such molecules. The clones utilized for the studies reported herein include clone 13367-1-5/C323, sub-clone 40 for Mincle, clone 13365-1-5/C374, sub-clone 10 for CD200 and clone 13366-1-2/C331, sub-clone 22 for CD200R. We cloned and sequenced the rhesus macaque homologues of human Mincle, CD200 and CD200R and the specificity of each of the selected mAb was defined by their reactivity against a series of overlapping peptides (12 mers overlapping by 6) that encoded the rhesus macaque homologues of human Mincle, CD200 and CD200R. The reactivity of the mAb utilized herein with specificity for rhesus macaque Mincle clone (13367-1-5/C323 subclone 40) was mapped to the “*pltkslsfwdvg”* sequence (a.a. residues 157–168), the reactivity of the mAb with specificity for rhesus macaque CD200 (clone 13365-1-5/C374 subclone 10) was mapped to the *“lhlgtvtdfkqt”* sequence (a.a. residues 217–228) and the reactivity of the mAb with specificity for rhesus macaque CD200R (clone 13366-1-2/C331 subclone 22) was mapped to the *“egaaqsnnslml”* (a.a. residues 25–36) sequence. Absorption of an aliquot of a known reactive titer of mAb with specificity for rhesus Mincle against a mixture of overlapping peptides encoding either Mincle, CD200, CD200R or for purposes of control irrelevant peptides encoding for SIVmac239, showed that the reactivity was only removed by absorption with the pool of peptides that encoded rhesus Mincle but not the other pools of peptides. Similarly, the reactivity of the mAb with specificity for rhesus CD200 and CD200R were only removed with pools of peptides that included the homologous peptides. Finally, the mAb selected failed to show reactivity against a variety of human lymphoid cell lines, primary hematopoietic rhesus macaque CD3+ T cells, B cells, and NK cells denoting specificity for the monocyte/macrophage lineages.

Each of these antibodies was biotinylated and used in conjunction with PE-avidin for defining their expression on cells from the 2 NHP species. For routine staining purposes 1 μg of each of the mAbs was used to stain a million PBMCs.

### Polychromatic Flow Cytometry

Cell populations following staining with the appropriate cocktail of antibody reagents were subjected to analysis using a BD LSR-Fortessa (BD Immunocytometry Division, Mountain View, CA). Data on a minimum of 10,000 events for lymphoid cells and 100,000 cells for mDC and pDCs were acquired and the data obtained subjected to analysis using the FlowJo Software (ver.9.6.2) (Ashland, OR). The gating strategies utilized for defining the various cell lineages has been published elsewhere [49] except for the monocyte lineage, which is outlined in detail herein.

### Virus Preparation

Stocks of SHIVBO159N4-p or desiv147C#4 viruses were prepared by infecting cell free virus from 293T sup to Concanavalin-A + IL-2 stimulated naïve rhesus macaque PBMC as previously described [50].

### Functional Analysis

Since the predominant cell lineage expressing these lectin-like molecules were determined to belong to the monocyte/macrophage series, studies of the function of these molecules were performed utilizing the monocyte tropic SHIV/SIV viruses and highly enriched populations of monocytes isolated from the PBMCs of normal rhesus macaques. The enriched population of monocytes was prepared from the peripheral blood mononuclear cells (PBMCs) following isolation from EDTA blood by Lymphoprep (Stemcell 07851) density-gradient centrifugation. Monocytes were separated from PBMCs using the CD14 monocyte enrichment kit (Easy Sep 19809) according to the manufacturer’s protocol. Briefly, cells were re-suspended at 0.5 x 10^6^ cells/ml in RPMI 1640 supplemented with 10% heat inactivated Fetal Bovine Serum (Hyclone SH30070.03), 1x Glutamax (Life Technologies REF 35050–061), 1x Gentamicin (Life Technologies REF 15750–060), 10 ng/ml GM-CSF (Sigma SRP3050) and 10 ng/ml M-CSF (Sigma M6518) and plated onto a 24 well Biocoat poly-D-lysine-coated plate (Corning 354414) in a volume of 1 ml and incubated at 37°C and 7% CO2 overnight to adhere monocytes. Non-adherent cells were then removed by washing several times with complete media and the adherent cells cultured with 1 ml complete media containing GM-CSF (10ng/ml) and M-CSF (10ng/ml) that was refreshed every other day for 9–12 days. When cells differentiated, each well was washed with complete media several times (3x). Controls included with each assay included cells cultured in media alone, cells cultured in the same media as utilized for growing virus stocks (mock infected) and cells cultured with media containing normal mouse IgG. The experimental cultures contained 0.5 ml RPMI-10 complete media with 1, 5 and 10 μg/ml of either monoclonal anti-CD200 (clone 13365-1-5/C374 sub-clone 10), anti-CD200R (clone 13366-1-2/C331 sub-clone 22) or anti-Mincle (clone 13367-1-5/C323 sub-clone 40) and incubated 2 hrs at 37°C. Subsequently, SHIVBO159N4-p or desiv147C#4 viruses were added (500 μl of virus containing 10^5^ TCID_50_/ml). Control wells received only virus and medium and the cultures were placed at 37°C overnight. At 24 hrs after incubation, cells were washed several times (3X) with complete media and maintained with one ml of complete RPMI media (for cell and virus control wells) or complete media with 1–10 μg/ml of the appropriate lectin monoclonal antibodies. Supernatant fluid were collected at (100 μl/well) every 3 days and replaced with equal amount of lectin like antibodies for a total of 20 days. SIV p27 Antigen Capture Assay (ABL cat # 5436) was performed on cell culture supernatant fluids as described by the manufacturer as a measure of virus quantitation. OD was recorded at 450 nm using Molecular Devices Vmax Kinetic Microplate Reader (Molecular Devices, Sunnyvale, CA). In addition, the specificity of the mAbs to inhibit virus replication was determined by pre-incubation of an aliquot of the anti-CD200 and anti-CD200R mAbs at 10 μg/ml with 5 μg/ml of the corresponding target peptides “*lhlgtvtdfkqt” and “egaaqsnnslml”*, respectively, or with an irrelevant peptide for 1 hr and subsequently each of these used in the inhibition assay as described above.

### Statistical analyses

Statistical significance was assessed using the two-tailed, unpaired *t* test or multiple comparisons. Statistical analyses were performed using GraphPad Prism (Version 6 for Mac OS X) Statistical software packages. P < 0.05 is represented by *; P < 0.001 is represented by ** and P < 0.0001 is represented by ***.

## Supporting Information

S1 Fig The gating strategy utilized for the analysis of rhesus CD200, CD200R and Mincle on the gated population of CD14+ cells. Aliquots of the samples are incubated in media containing an isotype control IgG or with a pre-determined optimal concentration of either biotinylated anti-CD200, CD200R or Mincle followed by PE-avidin and the profiles recorded.
(TIFF)

S2 Fig Representative flow cytometric profiles of Siglec-1 and Siglec-3 expression by the U937 (panel a), the THP-1 (panel b) cell lines and by PBMCs from a representative normal rhesus macaque (panel c). The red lines and numbers reflect the profile and MFI with the isotype control and the blue line the profile and MFI using the Anti-Siglec monoclonal antibodies, respectively.
(TIFF)

S3 Fig The gating strategy for the analysis of rhesus macaque plasmacytoid (pDCs) and myeloid dendritic cells (mDCs). Aliquots of PBMCs were stained with a cocktail of FITC-conj. lin+ antibodies and incubated either with a) PerCP 5.5 conj. anti-HLA-DR (clone G46-4), APC-conj. anti-CD11c (clone S-HCL3) and biotinylated anti-CD200, CD200R or Mincle followed by PE-avidin, or with b) Texas Red conj. anti-HLA-DR (clone G46-6), PerCP 55.5 conj. anti-CD123 (clone 7G3) and biotinylated anti-CD200, CD200R or Mincle followed by PE-avidin. The gated population of lin-, HLA-DR+ cells were then analyzed for CD11c or CD123 expression and the gated population of CD11c+/HLA-DR+ population (mDCs) and the gated population of CD123+/HLA_DR+ population (pDCs) analyzed for the frequencies of CD200, CD200R and Mincle expressing cells, respectively.
(TIFF)

S4 Fig Representative flow cytometric profiles of CD200, CD200R, and Mincle expressed by gated population of CD14+ of monocytes (prior to in vitro culture) and monocyte derived macrophages (post in vitro culture). Please note that the MFI for all 3 molecules is reduced but still readily detectable in the case of CD200 and CD200R.
(TIFF)
