# Supplementary figures and images for: Phenotypic and Functional Characterization of Monoclonal Antibodies with Specificity for Rhesus Macaque CD200, CD200R and Mincle
Source: PLoS One. 2015 Oct 15;10(10):e0140689. doi: 10.1371/journal.pone.0140689 (PMC4607400; doi:10.1371/journal.pone.0140689)

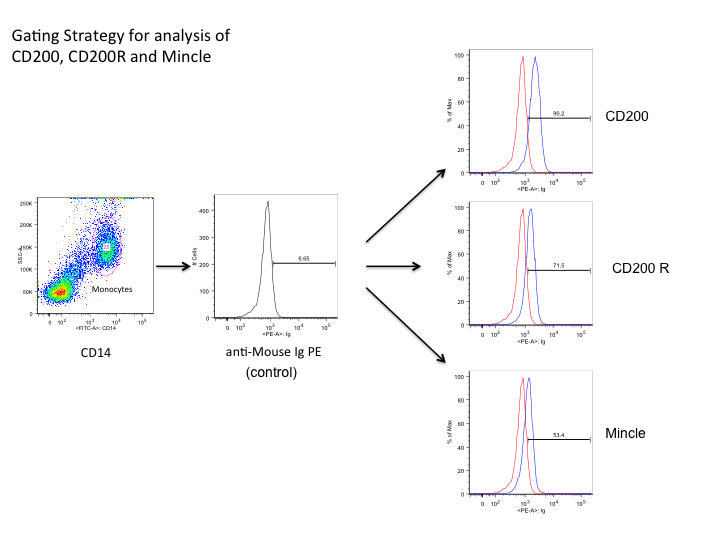

Supplement: S1 Fig — Aliquots of the samples are incubated in media containing an isotype control IgG or with a pre-determined optimal concentration of either biotinylated anti-CD200, CD200R or Mincle followed by PE-avidin and the profiles recorded. (TIFF) [file pone.0140689.s001.tiff]

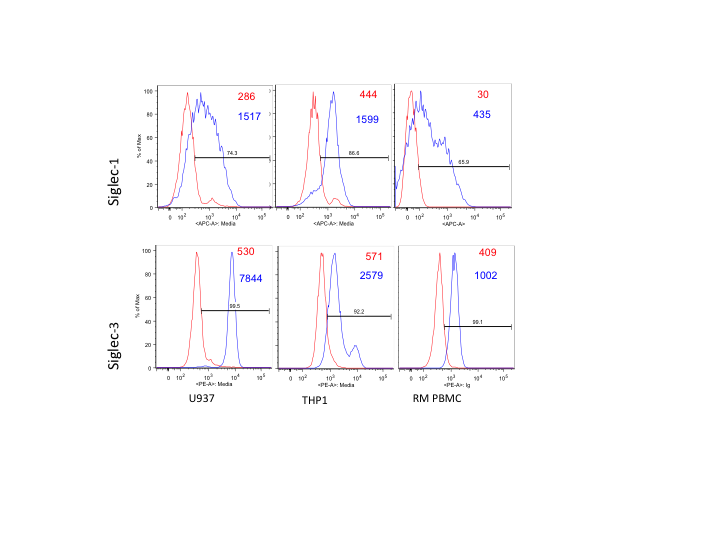

Supplement: S2 Fig — The red lines and numbers reflect the profile and MFI with the isotype control and the blue line the profile and MFI using the Anti-Siglec monoclonal antibodies, respectively. (TIFF) [file pone.0140689.s002.tiff]

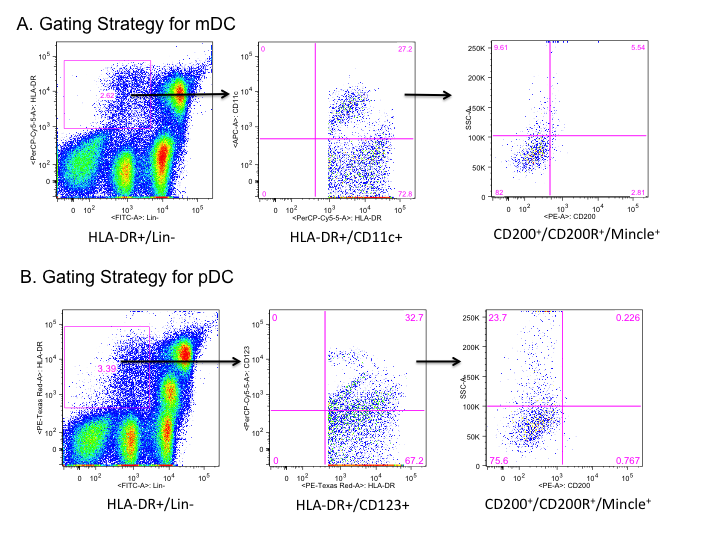

Supplement: S3 Fig — Aliquots of PBMCs were stained with a cocktail of FITC-conj. lin+ antibodies and incubated either with a) PerCP 5.5 conj. anti-HLA-DR (clone G46-4), APC-conj. anti-CD11c (clone S-HCL3) and biotinylated anti-CD200, CD200R or Mincle followed by PE-avidin, or with b) Texas Red conj. anti-HLA-DR (clone G46-6), PerCP 55.5 conj. anti-CD123 (clone 7G3) and biotinylated anti-CD200, CD200R or Mincle followed by PE-avidin. The gated population of lin-, HLA-DR+ cells were then analyzed for CD11c or CD123 expression and the gated population of CD11c+/HLA-DR+ population (mDCs) and the gated population of CD123+/HLA_DR+ population (pDCs) analyzed for the frequencies of CD200, CD200R and Mincle expressing cells, respectively. (TIFF) [file pone.0140689.s003.tiff]

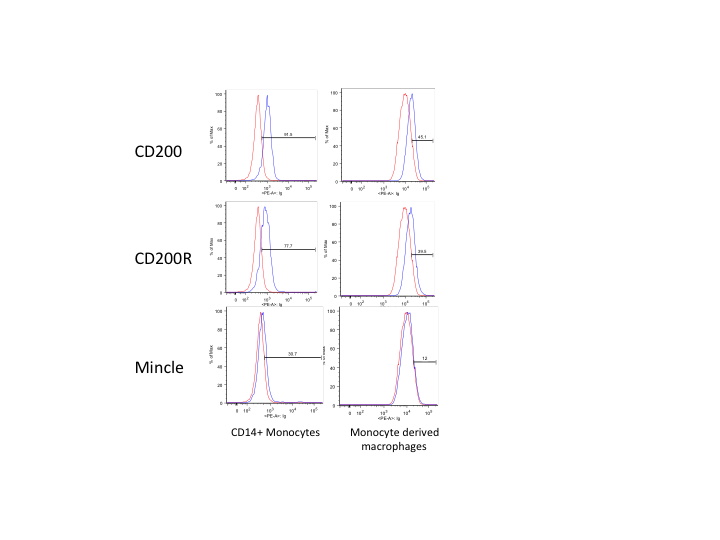

Supplement: S4 Fig — Please note that the MFI for all 3 molecules is reduced but still readily detectable in the case of CD200 and CD200R. (TIFF) [file pone.0140689.s004.tiff]
